# Death of Dr. Renfro

**Published:** 1898-07

**Authors:** 


DEATH OF DR. RENFRO.—Dr. J. C. B. Renfro, a prominent and well known physician, a long time practitioner at La Grange, Texas, died in Houston recently, of acute interstitial nephritis, the result of an attack of dengue last fall.

Dr. Renfro removed from La Grange, Texas, to Houston about two years ago, and at once entered upon an extensive general practice. After the attack of dengue, finding his health impaired, he removed back to La Grange, his old home, and entered into a copartnership with Dr. F. A. Schmitt, the well known physician and surgeon of that place, and they did a general practice under the firm name of Drs. Schmitt & Renfro.

Dr. Renfro was a graduate of the Medical Department of

Tulane University, New Orleans, of the class of 1872. He was a member of the Texas State Medical Association and also of the American Medical Association.

